# Relationships between selected indices of postural stability and sports performance in elite badminton players: Pilot study

**DOI:** 10.3389/fpsyg.2023.1110164

**Published:** 2023-03-22

**Authors:** Janusz Jaworski, Grzegorz Lech, Michał Żak, Kazimierz Witkowski, Paweł Piepiora

**Affiliations:** ^1^Faculty of Physical Education and Sport, University of Physical Education in Kraków, Krakow, Poland; ^2^Faculty of Physical Education and Sports, Wroclaw University of Health and Sport Sciences, Wrocław, Poland

**Keywords:** badminton, postural control, racket sports, visual information, different sports levels

## Abstract

The main aim of this study was to determine the relationships between postural stability and the place in the ranking of badminton players. The study examined 10 elite players from Polish national badminton team. The scope of the study included basic somatic characteristics, such as body height, body weight, BMI, and training experience. A Microgate GYKO inertial sensor system was used to assess the postural stability of athletes. Using Spearman’s rank correlation, cause-and-effect relationships between the place in the sports ranking and the analyzed variables characterizing postural stability were recognized. Depending on the distribution and homogeneity of variance, the significance of differences in variables that characterize postural stability between players of different sports skill levels (two groups) was calculated. The Student’s *t*-test or Mann–Whitney’s U-test was used for this purpose. In general, the athletes with higher positions on the ranking list presented a higher level of postural stability in both tests, which is also confirmed by the normalized values. However, for all variables of postural stability, no statistically significant correlations with sports ranking were observed. Higher values of Spearman’s rank correlation coefficients were found for the test performed in the one-foot standing test compared to the two-foot test. The results obtained indicate that particular attention in badminton training should be paid to the development of the level of postural stability in order to improve sports performance.

## Introduction

One of the most important coordination motor skills is the ability to maintain balance, which plays a key role because of the significant impact on maintaining a vertical body posture, as well as being essential when performing complex arbitrary movements (Woollacott and Shumway-Cook, 1990; Kubica et al., 2022). A high level of balance is needed in many sports, especially wherever open movement structures (dancing, figure skating, sport gymnastics, badminton, and judo) dominate (Coker and Kaminski, 2020; Slater et al., 2020; Gómez-Landero et al., 2021; Lu et al., 2022; Jaworski et al., 2023). It also determines the safe and independent performance of the basic and instrumental activities of daily living. It contributes to reducing the risk of falls, especially in older adults (Roeing et al., 2017; Cuevas-Trisan, 2019).

Static balance is most often defined as the ability to maintain the projection of the body’s center of gravity within the support area (Brachman et al., 2017). For the standing position, this means the area of foot contact with the ground including the surface area between them. From the biomechanical standpoint, it is defined as a state in which the net forces acting on the body are balanced and the sum of moments of these forces is zero (Pollock et al., 2000; Chiari and Cappello, 2005). In the standing position, a human is only seemingly in a state of equilibrium. This is because the human body constantly makes small corrective movements passing through the equilibrium point and moving away from it again. Maintaining balance is possible thanks to the processing of information from the following sensory inputs: from the vestibular organ (labyrinth), from visual, tactile, and proprioceptive systems. The information obtained is processed by the central nervous system and then transferred to the effector organs (Singh et al., 2012; Jaffri et al., 2017).

Another issue is the choice of tools and methods for measuring static and dynamic balance. In population studies (Topendsports, 2023),^1^ tests from three groups are most commonly used: Standing Balance (Flamingo Balance, Stork Stand Test, Standing Balance Test, One Leg Stand, and Stick Lengthwise Test), Walking Balance (Beam Walk, Balance Beam Test, Walk and Turn Field Sobriety Test), and Dynamic Balance (Balance Board Test, Bass Test, Star Excursion Balance Test, Y Balance Test, and Multiple Single-Leg Hop-Stabilization). Clinical trials, on the other hand, often use the following tests: Berg Balance Scale (BBS), Timed “Up & Go,” Single-leg stance test, 10-Meter Walk Test, BDL Balance Scale, Functional Reach test, Tinetti test, Mini Balance Evaluation Systems Test, and Unified Balance Scale (Lindmark et al., 2012; Takacs et al., 2014; Paillard and Noé, 2015; Hatfield et al., 2016; Bergquist et al., 2019; Kalkan et al., 2021). For many years, various types of balance platforms have been used to assess postural stability. They allow for the assessment of balance based on the displacements of the center of pressure (COP) on the support plane during free standing, which corresponds approximately to the projection of the center of gravity (COG) on the support plane (Błaszczyk, 2008; Lindmark et al., 2012). However, the platforms used have certain disadvantages: first of all, a high purchase price, which limits their widespread use, and they are usually large in size and complicated in use (Mancini et al., 2012). More advanced methods include computerized dynamic posturography on the NeuroCom Smart Equitest system® (Oregon, United States) and Biodex BioSway™ (Miner et al., 2020). Unfortunately, they are not widely used due to their large size, high purchase cost and much more complicated operation.

The above disadvantages and limitations forced the researchers to search for alternative tools to measure postural stability. It seems that different types of accelerometers can be an ideal solution. They are relatively inexpensive, small in size and do not require complicated software (Lindmark et al., 2012; Lesinski et al., 2016). The use of accelerometry (Bourke et al., 2010; Whitney et al., 2011; Marchetti et al., 2013) for recording body sway gained in popularity when the costs of accelerometers with improved measurement parameters declined and wireless technology became widespread. For these reasons, Microgate GYKO triaxial accelerometer was used in our research.

The importance of balance as an essential training element for badminton players, in preventing injuries and improving sports performance, has been highlighted by many authors. Moving around the court requires players to get to the shuttlecock as quickly as possible, while maintaining good balance and keeping the body under control. Malwanage et al. (2022) note the improvement of balance in young badminton players in 8 weeks of training. In the experiment, the control group performed 2 h of standard badminton training, while the experimental group additionally underwent 30 min of balance training, followed by 1 h and 30 min of regular training. Comparing the results before and after the experiment, it was found that both groups improved static balance (eyes open), but only the experimental group improved dynamic balance. On the other hand, a study by Erol (2022) observed the effectiveness of a basic badminton training program in children aged 11–12 on improving balance. The effect of plyometric training on dynamic balance and proprioception of the knee joint of female badminton players was sought in the work of Alikhani et al. (2019). The results of the study showed that a 6-week plyometric training program improved dynamic balance and knee proprioception in novice female badminton players. Investigating the effect of combined balance and plyometric training on the level of dynamic balance and performance of elite badminton players was sought in the work of Lu et al. (2022). Study participants were randomly divided into two groups. Both had the same technical training (badminton techniques for 6 days a week). One group performed balance training combined with plyometric training three times a week for 6 weeks (40 min of plyometrics and 20 min of balance training), while the other group performed only plyometric training (3–4 series × 8–12 repetitions for each exercise). The results obtained indicate that combined training is very promising in improving the dynamic balance and speed of elite badminton players. The necessity of including balance exercises in training programs for athletes of various sports is indicated by the review paper by Brachman et al. (2017). It was based on articles from PubMed and SportDiscus databases published between 2000 and 2016, and included articles on balance training, testing, and injury prevention in young, healthy athletes. In most of the articles analyzed, balance training was found to be an effective tool for improving postural control. However, it is not possible to establish a single training model that is suitable for every sport, as their specific nature and requirements must be taken into account.

The aim of this report was to determine possible relationships between selected indicators of postural stability and the sports ranking of elite badminton players. Furthermore, differentiation of postural stability indices was also sought, depending on the sports skill level of badminton players.

On the basis of the current state of knowledge in the addressed research problem, the following research hypotheses were adopted:

1. Cause-and-effect relationships will be observed between the athletes’ position in the sports ranking and selected indicators of postural stability.
2. Due to the nature of the game of badminton, higher correlation coefficients will be observed between the players’ position in the sports ranking and the postural stability test performed with one foot than in standing with both feet.
3. Higher levels of postural stability indices will be observed in athletes ranked higher in the sports ranking lists.
4. It is likely that no statistically significant differences in postural stability will be observed between the results of badminton players classified into two groups by sport level.

## Materials and methods

### Study group

The study group consisted of 10 males, players from the Polish National Badminton Team with an average training experience of 12.80 ± 2.74 years. In the 3 months prior to the study, all the athletes qualified for the study did not report any serious injuries, such as ankle or knee joint injury, chronic ligament dislocation, or other injuries to the lower limb. They also did not report neurological problems related to balance disorders. The research was carried out during the starting period during the players’ preparatory camp grouping. All badminton players participating in the training camp who consented to the study were covered. Thus, it was a purposive selection.

The observed population was a group of players competing in top-rank national and international tournaments. Analysis of the level of achievement of these badminton players reveals that all of them had won the highest trophies in national competitions, whereas some of them had participated in the Olympic Games and world championships; therefore, the competitors were elite Polish badminton players.

The tests and anthropometric measurements were performed in accordance with the Declaration of Helsinki. The examinations were approved by the Bioethics Committee at the Regional Medical Chamber in Kraków, Poland (approval No. 159/KBL/OIL/2017).

### Testing protocol

The participants performed all the tests barefoot, in the same room, between 11 a.m. and 2 p.m. Postural stability tests were conducted in a separate room providing peace and quiet for the badminton player being tested. The tests were conducted at a temperature of about 22°C providing thermal comfort. All postural stability tests were performed by the first author of the report. Before the test, the players did not perform warm-ups or other physical activity. During the examination, the athletes had their feet placed straight, with no rotation in the talocrural joint. Feet were spread to the width of the hips, whereas upper limbs were freely positioned along the torso. Immediately after the completion of this test, the contestants performed a one-legged test. They chose the dominant lower limb, whereas the other limb was bent in the knee joint at an angle of about 90°, with the upper limbs freely positioned along the torso. The Waterloo Footedness Questionnaire-Revised (WFQ-R) was used to determine the dominant limb (van Melick et al., 2017). During both tests, the athletes looked at the black point marked on the wall, 2 m away. The duration of each test was 30 s. During the first examination, we determined the height at which the GYKO system was to be attached. According to the manufacturer’s recommendations, this height should be set at the level of the T1 thoracic vertebrae (determined by palpation based on spinous processes). The tension of the GYKO attaching straps (chest circumference) was also adjusted to each player. The wireless transmission protocol was used to transfer data recorded by the GYKO inertial sensor to the laptop (Lenovo Yoga 500-15 i5-6200/8GB/1000/Win10).

### Scope of the study

Somatic characteristics were measured using the Martina technique. These included the following variables: body height (b-v), GYKO high (b-T1), and body mass (TANITA TBF-551 body composition analyzer). Measurements of somatic characteristics were taken by an experienced person employed by the “Motoric Laboratory.” Table 1 presents the basic statistical characteristics of age and selected somatic characteristics of the badminton players studied.

**Table 1:** Statistical characteristics of basic somatic parameters and age of the study participants.

Variable	*x¯*	SD	V[%]
Body height (cm)	180.80	6.07	3.35
Body mass (kg)	74.40	5.12	6.88
BMI (kg/m^2^)	22.78	1.65	7.24
GYKO height (cm)	149.10	8.56	5.74
Age (years)	22.27	4.64	20.83

The focus of the analysis was on the following variables that characterize postural stability (Gyko, 2022)^2^:

1. Area (mm^2^): The 95% ellipse of confidence is the ellipse that contains approximately 95% of the points of the trajectory.
2. Area Convex Hull (mm^2^): The Convex Hull is the smallest polygon that contains all the points of the trajectory.
3. Length: It is the total length of the trajectory obtained as the sum of the distances from one point to the next.
4. Length ML (medio-lateral; mm): The ML length is the total distance in the medio-lateral direction given as the sum of the absolute distances between two consecutive points in the ML direction.
5. Length AP (antero-posterior; mm): The AP length is the total distance in the anteroposterior direction given as the sum of the absolute distances between two consecutive points in the AP direction.
6. Mean Distance: This is the mean distance from the midpoint of the trajectory.
7. Mean Distance ML (mm): This is the mean distance from the midpoint of the medio-lateral trajectory.
8. Mean Distance AP (mm): This is the mean distance from the midpoint of the antero-posterior trajectory.
9. RMS Mean Distance: This is the dispersion of the distance (root mean square). In this case, as the points are centered on the mean, it is equivalent to the Standard Deviation.
10. RMS Distance ML (mm), AP (mm): This is the dispersion of the distance (root mean square). In this case, as the points are centered on the mean, it is equivalent to the Standard Deviation.
11. Mean Velocity: This is the mean travel velocity of the trajectory.
12. Mean Velocity ML (mm/s): This is the mean travel velocity of the trajectory in medio-lateral direction.
13. Mean Velocity AP (mm/s): This is the mean travel velocity of the trajectory in antero-posterior direction.

The sports skill level of the tested players was determined based on classification lists drawn up by the Polish Badminton Association (Polski Związek Badmintona, 2019; www.pzbad.pl).

### Statistical analysis

1. The Shapiro–Wilk test was used to test variables for normal distribution, whereas the Levene’s test was employed to assess the equality of variances.
2. The Spearman’s rank correlation coefficients between the postural stability indices and the place on the players’ ranking list were calculated (17).
3. The whole material was divided into two groups according to the sports skill level: high-level group (*n* = 5) and low-level group (*n* = 5). The basis for the division of players into two groups (depending on the sports level) was the current sports ranking (the list of the Polish Badminton Association) and the subjective classification of players proposed by the two coaches of the national team (purposive selection).
4. Depending on the distribution and homogeneity of variance, the significance of differences was calculated between groups with different sports skill levels. The Student’s *t*-test or Mann–Whitney’s U-test was used for this purpose. Furthermore, the effect size (Cohen, 1998) was also computed and interpreted as follows: ES > 0.2 = small, > 0.5 = medium, > 0.8 = large.
5. The size, range, and direction of differentiation of the tested indices of postural stability between the selected groups of badminton players were determined based on standardized differences. Standardization was performed for the group mean and standard deviation of the first group.

The calculations were performed using the STATISTICA 13.1 PL for Windows software package with the level of significance set at *p* ≤ 0.05. Furthermore, the effect size was determined by means of GPower 3.1 freeware, which is widely used in social studies (Faul et al., 2007).

## Results

The analysis will be started with Spearman’s rank correlation between the selected postural stability parameters and the place on the players’ ranking list. As results from Table 2, all correlation coefficients turned out to be statistically insignificant for the 2-ft standing test. It should be emphasized that in the adopted methodology, a positive sign of the correlation coefficient indicates the desired direction of relations between the analyzed variables, i.e., players classified higher on the ranking list had a higher level of postural stability indices. The athletes’ ranking showed the highest positive correlations with Convex Hull Area, Length, and Mean Velocity—correlation coefficients were about 0.30. Furthermore, negative correlation coefficients were obtained only for Mean Distance ML (−0.31) and RMS Distance ML (−0.13). This demonstrates that players with a worse position on the ranking list had higher results of both variables. Table 2 also shows correlation coefficients for the standing test performed on one leg. All correlation coefficients between the variables characterizing postural stability and the position on the ranking list were found positive. Therefore, the athletes with higher positions on the ranking list presented again a higher level of postural stability. The highest correlation coefficients (at the limit of statistical significance) were obtained for such variables as Area, Convex Hull Area, Mean Distance, and RMS Distance, and ranged from 0.41 to 0.50. Analysis of the rank correlation coefficients indicates that greater concurrence was observed between the place in the ranking and the results of the one-legged test compared to the 2-ft test.

**Table 2:** Spearman’s rank coefficients between selected postural stability parameters and the place of the player in the ranking.

Variable (Unit of measurement)	Two-feet test Spearman’s rho	Two-feet test 95% confidence interval (CI)	One-foot test Spearman’s rho	One-foot test 95% confidence interval (CI)
Area (mm^2^)	0.24	−0.46 ± 0.76	0.50	−0.19 ± 0.86
Convex hull area (mm^2^)	0.30	−0.41 ± 0.78	0.44	−0.26 ± 0.84
Length (mm)	0.31	−0.40 ± 0.79	0.38	−0.33 ± 0.81
Length AP (mm)	0.22	−0.48 ± 0.75	0.35	−0.36 ± 0.80
Length ML (mm)	0.26	−0.44 ± 0.76	0.41	−0.30 ± 0.83
Mean distance (mm)	0.18	−0.44 ± 0.76	0.43	−0.27 ± 0.83
Mean distance AP (mm)	−0.02	−0.62 ± 0.64	0.20	−0.49 ± 0.74
Mean distance ML (mm)	−0.31	−0.79 ± 0.40	0.15	−0.53 ± 0.71
RMS distance (mm)	0.12	−0.55 ± 0.70	0.43	−0.27 ± 0.83
RMS distance AP (mm)	−0.02	−0.64 ± 0.62	0.31	−0.40 ± 0.79
RMS distance ML (mm)	−0.13	−0.70 ± 0.54	0.25	−0.45 ± 0.76
Mean velocity (mm/s)	0.31	−0.40 ± 0.79	0.38	−0.33 ± 0.81
Mean velocity AP (mm/s)	0.22	−0.48 ± 0.75	0.35	−0.36 ± 0.81
Mean velocity ML (mm/s)	0.26	−0.44 ± 0.76	0.41	−0.30 ± 0.83

*Statistically significant correlation coefficients at *p* ≤ 0.05.

Basic statistical characteristics of parameters characterizing postural stability for the 2-ft test performed in groups depending on sports skill level are presented in Table 3. The results of the Student’s *t*-test revealed no statistically significant differences for all the variables characterizing postural stability. It is known that statistical significance depends on the effect size, but also on the sample size. Therefore, for a large sample, even a very small effect will be important. Taking into account the practical significance of the research, effect size should be documented simultaneously with the evaluation of the significance of differences, which is performed in this study. In our study, the greatest effect size (ES) of 0.71 was obtained for: Length AP (mm), Mean Velocity AP (mm/s), then for: Length (mm), Mean Velocity (mm/s)—0.59. The mean effect size was found for Convex Hull Area (mm^2^) and Area (mm^2^; ES of *ca.* 0.5). No effect size was found for variables: Mean Distance (mm), Mean Distance AP (mm), Mean Distance ML (mm), RMS Distance (mm), RMS Distance AP (mm), and RMS Distance ML (mm).

**Table 3:** Basic statistical characteristics of postural stability parameters, evaluation of the significance of intergroup differences and effect size (2-ft test).

Variable [unit of measurement]	Two-feet test high-level group		Two-feet test low-level group		*t*	*p*	z	ES
	arithmetic mean	SD	arithmetic mean	SD				
Area (mm^2^)	398.19	141.34	497.36	242.31	−0.79	0.45	−0.70	0.50
Convex hull area (mm^2^)	276.82	118.52	354.32	161.80	−0.86	0.41	−0.65	0.54
Length (mm)	217.09	43.12	247.85	59.77	−0.93	0.38	−0.71	0.59
Length AP (mm)	157.81	22.59	186.85	53.15	−1.12	0.29	−1.29	0.71
Length ML (mm)	115.84	37.53	125.87	26.78	−0.49	0.64	−0.27	0.30
Mean distance (mm)	7.74	1.32	7.61	2.70	0.10	0.92	0.10	0.06
Mean distance AP (mm)	6.73	1.81	6.51	2.66	0.15	0.89	0.12	0.09
Mean distance ML (mm)	2.93	0.83	2.87	0.74	0.11	0.91	0.07	0.07
RMS distance (mm)	8.75	1.30	8.76	3.21	−0.01	0.99	−0.01	0.00
RMS distance AP (mm)	7.94	1.69	8.00	3.23	−0.04	0.97	−0.04	0.02
RMS distance ML (mm)	3.45	0.91	3.45	0.91	−0.01	0,99	0.00	0.00
Mean velocity (mm/s)	10.85	2.16	12.39	2.99	−0.93	0.38	−0.71	0.59
Mean velocity AP (mm/s)	7.89	1.13	9.34	2.66	−1.12	0.29	−1.28	0.71
Mean velocity ML (mm/s)	5.79	1.88	6.29	1.34	−0.49	0.64	−0.27	0.30

*Statistically significant differences at *p* ≤ 0.05.

*t*, Student’s *t*-test value, U, Mann–Whitney U-test value (with continuity correction), *z*, standardized values, ES, effect size, > 0.2 = small, >0.5 = medium, >0.8 = large.

Regardless of the statistical analysis of the significance of mean differences, the analysis of standardized intergroup differences of analyzed parameters of postural stability provides interesting information. Such a methodological approach allowed for the analysis of the differentiation within all the tested properties (measured in different units). Analysis of the system of standardized differences revealed that a higher level of all parameters is presented by higher-ranked badminton players. In the case of the 2-ft standing test, the effect of the sports skill level on the results was the most pronounced: Length AP (mm), Mean Velocity AP (mm/s). A relatively large variation, in favor of the group with higher sports skill level, was also obtained for: Area (mm^2^), Length (mm), Mean Velocity (mm/s). The standardized values for these variables were *ca.* −0.70 SD.

Basic statistical characteristics of parameters characterizing postural stability for the one-foot test performed by both groups depending on sports skill level are presented in Table 4. The results of the Student’s *t*-test and Mann–Whitney’s U-test revealed no statistically significant differences for all the variables characterizing postural stability. However, a characteristic system of arithmetic means can be observed for all analyzed variables, with better results in the group with a higher sports skill level. According to the classification proposed by Cohen, the effect size for 12 variables should be considered medium (values of statistics ranged from 0.50 to 0.80). Table 4 also presents the standardized intergroup differences of the analyzed variables characterizing postural stability. Standardized group differences range from −0.11 SD to −1.00 SD. Analysis of the system of standardized differences revealed unequivocally that a higher level of all parameters is presented by higher-ranked badminton players (those with a higher position in the ranking). The largest normalized differences were obtained for variables: Length ML (mm) and Mean Velocity ML (mm/s).

**Table 4:** Basic statistical characteristics of postural stability parameters, evaluation of the significance of intergroup differences and effect size (one-foot test).

Variable (Unit of measurement)	One-foot test high-level group		One-foot test low-level group		*t*	*p*	z	ES
	arithmetic mean	SD	arithmetic mean	SD				
Area (mm^2^)	3128.04	2543.64	4817.60	2688.49	−1,02	0.34	−0.66	0.64
Convex hull area (mm^2^)	2211.98	1831.18	3349.38	1823.37	7.00^U^	0.29	−0.62	0.62
Length (mm)	620.63	203.18	809.30	355.69	−1.03	0.33	−0.93	0.65
Length AP (mm)	406.79	104.65	485.62	160.59	−0.92	0.38	−0.75	0.58
Length ML (mm)	381.28	159.80	541.29	303.11	9.00^U^	0.53	−1.00	0.66
Mean distance (mm)	17.82	7.87	21.83	7.65	−0.82	0.44	−0.51	0.51
Mean distance AP (mm)	11.24	5.93	14.84	8.15	−0.80	0.45	−0.61	0.50
Mean distance ML (mm)	11.45	5.82	12.10	5.13	−0.76	0.47	−0.11	0.11
RMS distance (mm)	19.72	8.57	24.14	8.38	−0.82	0.43	−0.52	0.52
RMS distance AP (mm)	13.50	6.83	18.02	9.54	−0.86	0.41	−0.66	0.54
RMS distance ML (mm)	13.67	7.19	14.64	5.85	−0.23	0.82	−0.13	0.14
Mean velocity (mm/s)	31.03	10.16	40.47	17.78	−1.02	0.33	−0.93	0.65
Mean velocity AP (mm/s)	20.34	5.23	24.28	8.03	−0.92	0.38	−0.75	0.58
Mean velocity ML (mm/s)	19.06	7.99	27.06	15.16	9.00^U^	0.53	−1.00	0.66

Symbols the same as in Table 3.

## Discussion

In sports training, the aim should be to recognize mutual cause-and-effect relationships between its type, somatic, energetic, coordination, and mental aptitudes and the development of the results achieved. The effectiveness of badminton playing depends on many combinations of factors which affect the player during the whole training process (Lees, 2003; Chansrisukot et al., 2015; Phomsoupha and Laffaye, 2015; Tomaszewski et al., 2018). An adequate level of coordination motor abilities is especially important in badminton. It is forced by the complex nature of the game, which requires the use of movement activities of high complexity and adaptation to constantly changing situations on the court (Wang et al., 2008, 2009; Poliszczuk and Mosakowska, 2009; Bańkosz et al., 2013; Jaworski and Żak, 2015; Kosack et al., 2020; Cui et al., 2022).

The high level of balance allows badminton players to use all their muscle strength and speed in a variety of categories of techniques such as smash, clear, and drop shot (Phomsoupha and Laffaye, 2015). During the game, players constantly follow the moving shuttlecock and quickly change their body position. The athletes attempt to maintain the projection of the body’s center of gravity (COG) within the support area by making very fast and asymmetrical movements of the upper limbs. On the other hand, after the action is completed, they have to quickly return to the correct starting position and prepare for the next play (Wong et al., 2019). A good balance also determines a more balanced landing on the ground after a jump, helps move faster on the court, and is an important factor in avoiding badminton injuries (Yung et al., 2007; Herbaut et al., 2018). The importance of balance for the playing performance in various positions (front court play, back court strokes, and jump smash). Therefore, improved body balance is critical for the development of movement skills in badminton and therefore determines high playing performance (Masu et al., 2014; Hamed and Hassan, 2017). The aim of the research was to fill the gap concerning the effect of balance on the playing performance of badminton players. Such reports have been very rare so far and therefore the authors point to the necessity of exploration of this area (Masu et al., 2014; Wong et al., 2019). The analysis of our results reveals positive correlations between selected parameters of postural stability and the position on the ranking list of badminton players. Much higher coefficients of correlation with the ranking for the one-foot test performed on the dominant limb were observed compared to the 2-ft standing test. As found by Wong et al. (2019) multi-plane movements, rapid changes in the player’s position, numerous jumps and lunges with the dominant lower limb, and the way the player moves around the court are specific to the game of badminton. Thus, it can be concluded that the results of the present study confirm this relationship. Badminton forces players to perform frequent jumps, sudden directional changes on the court, broad range of movements of the upper limbs, and frequent changes in body positions (Tiwari et al., 2011; Hu et al., 2015). For these reasons, the results of our research are obvious and likely to result from the nature of the dominant play in various unstable positions during competitions.

The analysis of the effective playing time revealed that the energy is largely fueled by aerobic pathways (around 60–70%), while around 30% of the energy is generated from anaerobic processes (Phomsoupha and Laffaye, 2015). The effect of fatigue on the results of dynamic balance in the Y balance test (YBT) was indicated by Sarshin et al. (2011). These authors found a decrease in the dynamic balance of the body after functional fatigue. For this reason, badminton players may be exposed to various injuries in the lower limbs. Similar findings were reported by Alikhani et al. (2019). Badminton players should be characterized by a high level of dynamic balance to prevent musculoskeletal injuries, especially non-contact anterior cruciate ligament (ACL) injuries. Therefore, badminton coaches and players can use plyometric training to improve dynamic balance, which in turn can reduce non-contact ACL injuries. Furthermore, Lu et al. (2022) also showed that balance training combined with plyometric training can enhance dynamic balance ability and improve the performance of male elite badminton players. The need for greater emphasis on stability training in junior badminton players was demonstrated by Vora et al. (2018). Understanding its importance in the overall improvement of sports performance is a must and can produce good results in the next stages of sports training.

Modern technological developments increasingly allow the use of various types of accelerometers (usually triaxial) to measure postural stability. Particularly after the cost of such tools was reduced and wireless technology was used, they became popular in scientific research. The ICCs reliability results of postural stability measurements obtained with accelerometers are good and are usually above 0.75 (Marchetti et al., 2013; Saunders et al., 2015; Guo et al., 2022). The GYKO accelerometer (Microgate Italy) used in our study has high reliability and accuracy of measurement. It has been used in a number of studies that have looked at various aspects of human motor skills (Lesinski et al., 2016; Arede et al., 2019; Santospagnuolo et al., 2019; Hamersma et al., 2020). In a study by Jaworski et al. (2020), the authors determined the reliability of a GYKO accelerometer. The results that characterize postural stability indices showed high and satisfactory values of intraclass correlation coefficients (ICCs) between test and retest data (ICCs values ranging from 0.62 to 0.70).

So far, most research has been devoted to the comparison of the level of selected coordination skills between athletes practicing different sports and non-athlete peers and athletes at different sports skill levels. In this area of research, the greatest achievements concern comparisons of reaction time. The review of the results indicates that badminton players had shorter reaction times compared to those from non-athlete control groups (Bańkosz et al., 2013; Dube et al., 2015). Furthermore, Yüksel and Tunç (2018) demonstrated that the reaction times of young badminton players from the highest-ranked countries were better. Wong et al. (2019) compared the level of dynamic and static balance in badminton and control group players, without any significant differences between the groups. These results are slightly different from other studies indicating that badminton training can improve balance. The authors explain this by the age of the players surveyed, who had already developed an almost mature postural control system, so the potential for further improvement could be limited. Furthermore, the control group consisted of physically active individuals, which probably had a positive effect on the level of their balance abilities.

In our study, we also compared selected variables characterizing postural stability between badminton players with different sports skill level. For both analyzed samples, the differences in mean results were statistically insignificant. However, analysis of the system of standardized differences revealed that a higher level of all parameters was presented by players from the group with a higher sports skill level. The calculated Cohen effect size should be regarded as medium for most variables. These regularities are particularly noticeable for the test performed on the dominant limb. Research on the displacements of the center of gravity (COG) of eight high-level athletes (belonging to the top three teams of the Badminton Championship in Japan) and eight amateur badminton players playing in university clubs was carried out by Masu et al. (2014). In the test with eyes open, the COG was maintained in the high-level group close to the center, while the low-level group moved it more toward the dominant leg. In the test with eyes closed, the length of the statokinesiogram path, the sway area, and the amplitude of sway in the X and Y axes were larger in the group with lower sports skill level. The quoted results are consistent with our findings. Furthermore, Yüksel et al. (2015) demonstrated that the 8-week training of young badminton players improves dynamic balance. Similar conclusions were presented by Masu et al. (2014), who stated that training can improve static balance in standing on one limb with eyes closed. Therefore, the observed results may have been caused by long-term physical training, which leads to specific and plastic changes in the central nervous system (Masu et al., 2014).

We believe that in the training programs of badminton players, special attention should be paid to the formation of balance. The training structure should include various types of static and dynamic balance exercises. Balance exercises should take into account different positions (one-legged, two-legged, and tandem), the ground (stable, unstable), and be conducted with eyes open and with the removal of visual feedback.

### Limitation of the study

It is necessary to study the effects of basic postural stability training in badminton players of different training seniority as well as sports level. Such studies should be conducted in different age groups in both sexes.

Further studies are needed in groups with much larger numbers of individuals.

Postural stability studies should be conducted with consideration of different starting positions, with eyes open or closed and on a stable or unstable surface.

The use of alternative measurement tools for assessing balance should also be considered (stabilometric platforms, stabilographic single-plate or dual-plate versions, and balance boards).

## Conclusion

The results presented in the study lead to the following conclusions:

1. Spearman’s rank correlation coefficients indicate the cause-and-effect relationships between the ranking of badminton players and postural stability indices. These relationships are particularly noticeable for the one-foot test performed on the dominant limb.
2. A higher level of postural stability is observed by badminton players classified higher on the ranking lists.
3. The results obtained indicate that particular attention in badminton training should be paid to the development of the level of postural stability in order to improve sports performance.
4. Further research should be conducted for different training groups and sports skill levels in order to confirm the effect of balance training on the effectiveness of playing badminton.

## Data availability statement

The original contributions presented in the study are included in the article/supplementary material, further inquiries can be directed to the corresponding author.

## Ethics statement

The studies involving human participants were reviewed and approved by Bioethics Committee at the Regional Medical Chamber in Kraków, Poland (Approval No. 159/KBL/OIL/2017). The patients/participants provided their written informed consent to participate in this study.

## Author contributions

JJ, GL, and MŻ contributed to conception and design of the study. JJ and MŻ organized the database. JJ and GL performed the statistical analysis. JJ, KW, and PP wrote the first draft of the manuscript and wrote the sections of the manuscript. All authors contributed to the article and approved the submitted version.

## Conflict of interest

The authors declare that the research was conducted in the absence of any commercial or financial relationships that could be construed as a potential conflict of interest.

## Publisher’s note

All claims expressed in this article are solely those of the authors and do not necessarily represent those of their affiliated organizations, or those of the publisher, the editors and the reviewers. Any product that may be evaluated in this article, or claim that may be made by its manufacturer, is not guaranteed or endorsed by the publisher.

## Footnotes

^1^ https://www.topendsports.com

^2^ www.gyko.it
